# Minimization of Resource Consumption with URLLC Constraints for Relay-Assisted IIoT

**DOI:** 10.3390/s25154846

**Published:** 2025-08-06

**Authors:** Yujie Zhao, Tao Peng, Yichen Guo, Yijing Niu, Wenbo Wang

**Affiliations:** School of Information and Communication Engineering, Beijing University of Posts and Telecommunications, Beijing 100876, China; yjzhao@bupt.edu.cn (Y.Z.); guoyichen@bupt.edu.cn (Y.G.); silhouette@bupt.edu.cn (Y.N.); wbwang@bupt.edu.cn (W.W.)

**Keywords:** uRLLC, relay-assisted communications, finite blocklength, resource consumption minimization

## Abstract

In relay-assisted Industrial Internet of Things (IIoT) systems with ultra-reliable low-latency communication (uRLLC) requirements, finite blocklength coding imposes stringent resource constraints. In this work, the packet error probability (PEP) and blocklength allocation across two-hop links are jointly optimized to minimize total blocklength (resource consumption) while satisfying reliability, latency, and throughput requirements. The original multi-variable problem is decomposed into two tractable subproblems. In the first subproblem, for a fixed total blocklength, the achievable rate is maximized. A near-optimal PEP is first derived via theoretical analysis. Subsequently, theoretical analysis proves that blocklength must be optimized to equalize the achievable rates between the two hops to maximize system performance. Consequently, the closed-form solution to optimal blocklength allocation is derived. In the second subproblem, the total blocklength is minimized via a bisection search method. Simulation results show that by adopting near-optimal PEPs, our approach reduces computation time by two orders of magnitude while limiting the achievable rate loss to within 1% compared to the exhaustive search method. At peak rates, the hop with superior channel conditions requires fewer resources. Compared with three baseline algorithms, the proposed algorithm achieves average resource savings of 21.40%, 14.03%, and 17.18%, respectively.

## 1. Introduction

Ultra-reliable low-latency communication (uRLLC) is a key component of current fifth-generation (5G) and future sixth-generation (6G) mobile communication networks [1]. URLLC enables mission-critical industrial applications by ensuring 99.999% reliable delivery of a 32-byte packet within 1 millisecond [2]. However, in wireless networks, blockages and fading hinder reliable transmission. Recent studies have shown that relay is one of the effective means to improve transmission quality and guarantee reliable communication by reducing path loss and providing spatial diversity [3,4].

Relay-assisted communications have been widely concerned in the past years. The communications in the infinite blocklength regime and finite blocklength (FBL) regime were considered in [5,6] and [7,8,9,10,11,12], respectively. Bao et al. [5] analyzed the performance under amplify-and-forward (AF) and decode-and-forward (DF) relays. The received signals from both the direct link and indirect link were combined at the receiver by the maximal ratio combining (MRC). In [6], the receiver decoded the packet based on the accumulated mutual information (MI) of the direct link and indirect link. In [7,8,9,10,11,12], finite blocklength coding is employed to achieve low-latency transmission. Finite blocklength coding reduces latency by using shorter codewords, thereby shortening transmission and processing time. In [7,8,9], it was assumed that the blocklength was equal in the two hops. Singh et al. [7] proposed an energy-efficient resource allocation algorithm to jointly optimize the transmit power, sub-carrier allocation, and error probability. Cheng et al. [8] enabled uRLLC transmission while minimizing transmission power by jointly optimizing relay selection, resource block (RB) assignment, and transmit power allocation. Kurma et al. [9] considered a full-duplex two-way communication system and developed an adaptive AF/DF relaying protocol. Additionally, exact closed-form expressions for the outage probability were derived. In [10], the authors investigated blocklength allocation in two-hop communication to maximize information rate. In [11,12], the packet error probability (PEP) is minimized by optimizing blocklength allocation in two-hop communication. After certain approximations and relaxations, iterative algorithms based on convex optimization are introduced in [10,11,12]. It should be noted that most studies in the FBL regime, such as [7,8,9,10,11], assumed that there was no direct link between the transmitter and receiver.

By reviewing the research on uRLLC-enabled relay-assisted systems in the FBL regime, we can find that there are still aspects of the current research that require further attention. Firstly, existing works, such as [9,11,12], generally concentrate on improving PEP. In IIoT scenarios, the increasing demand for uRLLC exacerbates wireless resource shortages. However, there is still a lack of research aimed at maximizing resource utilization or minimizing resource consumption, which is particularly significant in wireless communication scenarios with limited resources. The resource consumption is influenced by PEP and blocklength allocation between the two hops. Secondly, though some of the current research, for example, references [10,11,12], explored blocklength allocation in two-hop communication, most were based on iterative solutions. The closed-form solution is still lacking for optimal blocklength allocation, and less consideration has been given to the effect of PEP allocation on the transmission performance. Finally, the current literature lacks systematic evaluation for the necessity of relay activation. Since an accurate signal-to-interference-plus-noise ratio (SINR) or signal-to-noise ratio (SNR) for each link can be obtained by using the method proposed in our previous work [13], solving the aforementioned problems is meaningful and feasible.

This paper considers a simplified uplink Industrial Internet of Things (IIoT) scenario, which includes a source node, a half-duplex DF relay node, and a destination node. The source node needs to transmit a small packet to the destination node under uRLLC requirements. There are two selectable communication modes. In the direct communication (DC) mode, the source node sends packets via direct link. While in the relay communication (RC) mode, the relay node is enabled to forward the packets. The received signals are combined at the destination node. Limited transmission power at the source and relay nodes is considered. Corresponding to the above analysis, the main contributions of this paper can be summarized as follows.

1. Unlike the recent studies that mainly focus on PEP minimization, this work aims to minimize resource consumption, which is the total blocklength of the two hops, by optimizing the PEPs of each hop and the blocklength allocation between the source and relay nodes. Latency, reliability, and coding rate constraints are taken into account. This is equivalent to maximizing resource utilization, which is meaningful in resource-limited IIoT scenarios.
2. The original problem of resource consumption minimization is reformulated as two equivalent subproblems. In the first subproblem, for a given total blocklength, we consider the joint optimization of PEP and blocklength allocation for achievable rate maximization (JPB-ARM). The expressions of the objective function and constraints of the first subproblem vary under different communication modes. Therefore, an optimal solution is found for each mode, and the mode with the higher achievable rate is selected. Under the RC mode, through theoretical analysis, a near-optimal solution for the PEPs of each hop is first obtained. Then, the first subproblem is reformulated as a quartic equation, and the closed-form solution for optimal blocklength allocation is derived using Ferrari’s method. Under the DC mode, the maximum achievable rate of the system is derived through a simple proof. In the second subproblem, the resource consumption minimization (RCM) is considered, which is solved by integrating the solution of the first subproblem with the bisection search method.

The rest of the paper is organized as follows. In Section 2, the system model is introduced and the problem of resource consumption minimization is formulated. Section 3 presents the solution to the minimizing resource consumption problem in detail. Section 4 shows the simulation results and analysis, followed by the conclusion of the paper in Section 5. Table 1 lists the main variables used in this study along with their corresponding meanings.

## 2. System Model and Problem Formulation

### 2.1. System Model

**Figure 1:**
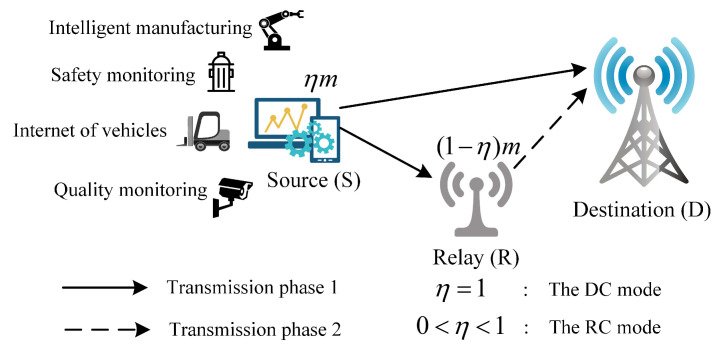
System model of the relay-assisted IIoT network.

In certain IIoT communication scenarios, such as intelligent manufacturing, safety monitoring, internet of vehicles, and quality monitoring, the transmission of sensor data (or control instructions) is generally subject to the stringent requirements of uRLLC. In wireless networks, the link conditions vary significantly across different transmitter–receiver pairs. For transmitter–receiver pairs suffering from blockage or severe channel fading, it is challenging to ensure uRLLC transmission. Using relay nodes can mitigate these issues and improve communication performance by providing spatial diversity. By optimizing the blocklength allocation and PEPs for the two hops based on the channel conditions, the uRLLC transmission performance can be further ensured. In this work, a simplified uplink IIoT communication scenario with a source (S) node, a half-duplex DF relay (R) node, and a destination (D) node is considered, as shown in Figure 1 (The application of the proposed scheme to multi-user and interference scenarios is discussed in Section 4.3).

In the IIoT scenarios, numerous devices are present, each with different activity periods. Some of them are not transmitting data at the current time, which are called idle devices and can act as R nodes (In IIoT scenarios, low-cost devices are typically equipped with a single antenna, and face challenges in implementing complex relay technologies). S is an active device that needs to transmit a small packet to D. D is a base station. The packet size is denoted as *k* bits. To support industrial application, reliability constraint is taken into account, i.e., the PEP should be no more than $\varepsilon_{max}$. In addition, a latency constraint is considered, i.e., the transmission should be finished within $m_{max}$ symbols or channel uses. Correspondingly, the communication time does not exceed $T_{comm}=m_{max}T_{s}$ milliseconds, where $T_{s}$ is the symbol duration (In this work, our primary focus is on the blocklength allocation, and thus we concentrate on the communication delay, $T_{comm}$. The relay activation delay and processing delay are assumed to be constant and collectively denoted as $T_{other}$. Therefore, under a total end-to-end latency constraint $T_{all}$, $T_{comm}=T_{all}-T_{other}$). $T_{comm}$ is typically no more than 1 millisecond [2], which is much shorter than the channel coherence time. Hence, we consider that the channels are quasi-static fading and remain constant during the transmission of a packet and change in the next packet transmission [8,11,12].

S, R, and D communicate over complex Gaussian channels. The channel fading coefficients of the S-D, S-R, and R-D links are denoted as $h_{SD}\in C$, $h_{SR}\in C$, and $h_{RD}\in C$, respectively. Then, the SNRs of the S-D, S-R, and R-D links are $\gamma_{SD}=\frac{{\left|h_{SD}\right|}^{2}p_{S}}{\sigma^{2}}$, $\gamma_{SR}=\frac{{\left|h_{SR}\right|}^{2}p_{S}}{\sigma^{2}}$, and $\gamma_{RD}=\frac{{\left|h_{RD}\right|}^{2}p_{R}}{\sigma^{2}}$. $\sigma^{2}$ is the additive white Gaussian noise variance. $p_{S}$ and $p_{R}$ are the constant transmit powers at S and R, respectively. $\gamma_{SD}$, $\gamma_{SR}$, and $\gamma_{RD}$ can be accurately estimated by using the method proposed in our previous work [13].

In contrast to other studies that assume relays are always employed, this work dynamically decides whether to activate R depending on $\gamma_{SD}$, $\gamma_{SR}$, and $\gamma_{RD}$. Therefore, in this work, two selectable communication modes, the RC and DC modes, are considered. R is active only when the RC mode can provide better communication performance than the DC mode. Suppose S and R jointly occupy *m* channel uses to transmit the packet, $m\leq m_{max}$. This work aims to achieve optimal blocklength and PEP allocation between the two hops with the objective of minimizing resource consumption, *m*. The two communication modes, along with the research objectives, are described in detail below.

### 2.2. Achievable Rate Under the RC Mode in the FBL Regime

To satisfy the transmission latency constraint, FBL codes are adopted. Under the RC mode, the transmission is divided into two phases. The blocklength should be allocated between S and R, and the PEP of the two hops should be optimized. Let η denote the proportion of the total blocklength occupied by S. Then the proportion of the total blocklength occupied by R is 1−η. η satisfies 0<η<1.

In the first transmission phase, S broadcasts a data packet with ηm channel uses. Shannon’s capacity formula is intended for infinite blocklengths and is not applicable to the considered FBL codes scenarios. By using the finite blocklength rate formula derived in [14], the achievable rate of the S-R link (in bits per channel use) can be represented as

(1) $$\begin{array}{c} R_{SR}\left(m,\eta ,\varepsilon_{SR}\right)=\eta \left(C_{SR}-\sqrt{\frac{V_{SR}}{\eta m}}\frac{Q^{-1}\left(\varepsilon_{SR}\right)}{\ln2}\right), \end{array}$$

where $C_{SR}=\log_{2}\left(1+\gamma_{SR}\right)$. $\varepsilon_{SR}$ is the PEP for the S-R link. $V_{SR}=1-\frac{1}{{\left(1+\gamma_{SR}\right)}^{2}}$ is the channel dispersion. $Q^{-1}\left(\cdot \right)$ is the inverse of Gaussian Q-function, which is denoted by $Q\left(x\right)=\frac{1}{\sqrt{2\pi }}\int_{x}^{\infty }e^{-\frac{t^{2}}{2}}dt$.

If R successfully decodes the data packet, it will forward the data packet to D with the remaining (1−η)m channel uses in the second transmission phase. The S-D and R-D links together form a combined link, and D combines the received signals. According to [15], the achievable rate of the combined link can be represented as

(2) $$\begin{array}{c} R_{C}\left(m,\eta ,\varepsilon_{C}\right)=C_{C}-\sqrt{\frac{V_{C}}{m}}\frac{Q^{-1}\left(\varepsilon_{C}\right)}{\ln2}, \end{array}$$

where $C_{C}=\eta C_{SD}+\left(1-\eta \right)C_{RD}$, $V_{C}=\eta V_{SD}+\left(1-\eta \right)V_{RD}$. $C_{XD}=\log_{2}\left(1+\gamma_{XD}\right)$, $V_{XD}=1-\frac{1}{{\left(1+\gamma_{XD}\right)}^{2}}$ with X∈{S,R}. $\varepsilon_{C}$ is the PEP of the combined link.

The relay-assisted communication is limited by the bottleneck link, either the S-R link or the combined link. Therefore, the achievable rate under the RC mode is calculated as

(3) $$\begin{array}{c} R_{relay}\left(m,\eta ,\varepsilon_{SR},\varepsilon_{C}\right)=\min\left\{R_{SR}\left(m,\eta ,\varepsilon_{SR}\right),R_{C}\left(m,\eta ,\varepsilon_{C}\right)\right\}. \end{array}$$

System decoding is correct only when both the first and second phase decodings are correct. Therefore, the overall PEP requirement can be described as

(4) $$\begin{array}{c} \varepsilon_{R}=1-\left(1-\varepsilon_{SR}\right)\left(1-\varepsilon_{C}\right)\approx \varepsilon_{SR}+\varepsilon_{C}\leq \varepsilon_{max}. \end{array}$$

$\varepsilon_{max}$ is the PEP limit, which usually satisfies $\varepsilon_{max}\leq 10^{-5}$ in IIoT. The approximation is accurate enough since $\varepsilon_{SR}$ and $\varepsilon_{C}$ are extremely small.

### 2.3. Achievable Rate Under the DC Mode in the FBL Regime

S occupies *m* channel uses to send packets to D via direct link, while R does not occupy any resources. In this mode, η=1. The achievable rate is calculated as

(5) $$\begin{array}{c} R_{D}\left(m,\varepsilon_{D}\right)=C_{SD}-\sqrt{\frac{V_{SD}}{m}}\frac{Q^{-1}\left(\varepsilon_{D}\right)}{\ln2}. \end{array}$$

$\varepsilon_{D}$ is the PEP under the DC mode, which should satisfy $\varepsilon_{D}\leq \varepsilon_{max}$.

### 2.4. The System’s Achievable Rate in the FBL Regime

As mentioned earlier, the communication mode with higher achievable rate is chosen. Therefore, the achievable rate of the system is calculated as

(6) $$\begin{array}{cc} R_{system}\left(m,\eta ,E\right) & =\max\{R_{relay}\left(m,\eta ,\varepsilon_{SR},\varepsilon_{C}\right),R_{D}\left(m,\varepsilon_{D}\right)\}. \end{array}$$

$E=\{\varepsilon_{D},\varepsilon_{SR},\varepsilon_{C}\}$ is the set of PEPs. For any given values of m,η, and E representing a particular blocklength and PEP allocation scenario, $R_{relay}$ and $R_{D}$ can be derived using (3) and (5), respectively. The communication mode, which offers a higher achievable rate, is selected. Different values of m,η, and E correspond to different optimal communication modes and system rates. It should be noted that for a given η∈(0,1), if $R_{relay}<R_{D}$, η is set to 1. Because, when using the DC mode, η remains constant at 1.

**Figure 2:**
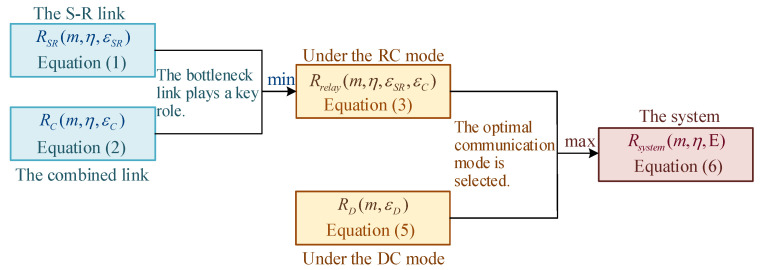
The relationship between the achievable rates under different communication modes, as defined in Equations (1), (2), (3), (5) and (6).

Since several equations for calculating the achievable rate have been introduced above, Figure 2 is provided to intuitively show their relationships.

### 2.5. The Problem of Minimizing Resource Consumption

This work aims to minimize overall blocklength overhead, *m*, by optimizing the PEPs of each link and the blocklength allocation between S and R, which is formulated as:

$$\begin{array}{cc} (P0): & \;\min_{m,\eta ,E}\;\;m\\ s.t.\;\; & C1:\left\lfloor \eta \right\rfloor \varepsilon_{D}+\left(1-\left\lfloor \eta \right\rfloor \right)\left(\varepsilon_{SR}+\varepsilon_{C}\right)\;\;\leq \varepsilon_{\max}\\ \; & C2:m\leq m_{\max}\\ \; & C3:R_{system}\left(m,\eta ,E\right)\geq \frac{k}{m}\\ \; & C4:0<\eta \leq \;1\\ \; & C5:m,\eta m\in N^{+}. \end{array}$$

$\left\lfloor \eta \right\rfloor$ represents the floor function applied to η. Under the RC mode, $\left\lfloor \eta \right\rfloor =0$ with 0<η<1. Under the DC mode, $\left\lfloor \eta \right\rfloor =1$ with η=1. Constraint C1 and C2 limit the reliability and communication delay. Constraint C3 limits the coding rate. the packet of *k* bits is transmitted with *m* blocklength, in either the RC or the DC mode. The resulting coding rate equals $\frac{k}{m}$, which should be less than $R_{system}$. Constraint C4 indicates that S must utilize resources to transmit, while the transmission of R is optional. Constraint C5 is the non-negative integer constraint of blocklength, where $N^{+}$ denotes the positive integer set.

Adopting relay causes each hop to occupy fewer resources than direct communication, potentially resulting in performance degradation. However, utilizing a relay can offer advantages when its channel conditions surpass those of the direct link. Therefore, the optimal communication mode, η, *m*, and E are related with channel conditions, $\gamma_{SD}$, $\gamma_{SR}$, and $\gamma_{RD}$.

## 3. The Solution of Problem (P0)

### 3.1. An Equivalent Formulation of Problem (P0)

The optimization of Problem (P0) involves multiple variables, including m,η, and E, and is intractable in its original form. To reduce the computational complexity, the original problem is decomposed into two subproblems (P1) and (P2), thereby reducing the number of variables to be optimized in each. First, given $m=m_{0}\in N^{+}$, we consider the JPB-ARM problem (Problem (P1)). We begin with Problem (P1), since it shares an underlying objective with Problem (P0)—improving resource utilization. Problem (P1) is formulated with a given *m*, while Problem (P2) aims to determine its minimum value. This decomposition simplifies the problem-solving process without altering the underlying optimization logic or objective.

Problem (P1) is formulated as:

$$\begin{array}{cc} (P1): & \;\max_{\eta ,E}\;\;R_{system}\left(m_{0},\eta ,E\right)\\ s.t.\;\; & C1,C4,\\ \; & C5-1:\eta m_{0}\in N^{+}. \end{array}$$

The solution of Problem (P1) is denoted as $\eta_{system}^{*}\left(m_{0}\right)$ and $E_{\mathrm{system}}^{*}\left(m_{0}\right)$. Correspondingly, the optimal value of $R_{system}$ for a given $m_{0}$ is $R_{system}\left(m_{0},\eta_{system}^{*}\left(m_{0}\right),E_{\mathrm{system}}^{*}\left(m_{0}\right)\right)$, which is denoted as $R_{system}^{*}\left(m_{0}\right)$ for simplicity.

Each value of $m\in N^{+}$ corresponds to a maximum achievable rate $R_{system}^{*}\left(m\right)$. Next, we consider the RCM problem. The goal is to minimize *m* for completing the transmission of a *k*-bit data packet, described as Problem (P2):

$$\begin{array}{cc} (P2): & \;\min_{m}\;\;m\\ s.t.\;\; & C2,\\ \; & C3-2:R_{system}^{*}\left(m\right)\geq \frac{k}{m},\\ \; & C5-2:m\in N^{+}. \end{array}$$

Then, the following proposition can be derived:

**Proposition** **1.** $R_{system}^{*}\left(m\right)$ increases monotonically with respect to m. If $m_{max}R_{system}^{*}\left(m_{max}\right)\geq k$, the Problem (P1) and (P2) are feasible. And the optimal solution to Problem (P1) and (P2) coincides with that to Problem (P0).

**Proof of Proposition** **1.** Please see Appendix A.  □

### 3.2. Analysis of Problem (P1) and (P2)

To solve the optimization Problem (P1) and (P2), the following proposition is first provided.

**Proposition** **2.** *Constraints C1 of Problem (P1) holds with equality at the optimum solution.*

**Proof of Proposition** **2.** Please see Appendix B.  □

Problem (P1) must be solved as a prerequisite to addressing Problem (P2). In Problem (P1), as Constraint C1 involves different conditions under two communication modes, separate analysis is required.

1. Under the DC mode, $R_{system}\left(m_{0},\eta ,E\right)=$$R_{D}\left(m_{0},\varepsilon_{max}\right)$ with η=1, and $\varepsilon_{D}=\varepsilon_{max}$ according to Proposition 2. The value of $R_{D}\left(m_{0},\varepsilon_{max}\right)$, or denoted simply as $R_{D}^{*}\left(m_{0}\right)$, is determined, because $m_{0}$ and $\varepsilon_{max}$ are known.
2. Under the RC mode, $R_{system}\left(m_{0},\eta ,E\right)=$$R_{relay}\left(m_{0},\eta ,\varepsilon_{SR},\varepsilon_{C}\right)$. Based on Proposition 2, Constraint C1 is transformed into $\varepsilon_{SR}+\varepsilon_{C}=\varepsilon_{\max}$. In addition, Constraint C4 is transformed into 0<η<1. In fact, the feasible range of η can be relaxed to (0,1] under the RC mode. This does not affect the final outcome of Problem (P1), which is demonstrated in Appendix C. When $m_{0}$ is known, the optimization of $\varepsilon_{SR}$, $\varepsilon_{C}$, and η is required to obtain the maximum value of $R_{relay}$, which is formulated as:
  $$\begin{array}{cc} (P1-1): & \;\max_{\eta ,\varepsilon_{SR},\varepsilon_{C}}\;\;R_{relay}\left(m_{0},\eta ,\varepsilon_{SR},\varepsilon_{C}\right)\\ s.t.\;\; & C1-1:\varepsilon_{SR}+\varepsilon_{C}\;\;=\varepsilon_{\max}\\ \; & C4,C5-1. \end{array}$$
  The optimal value of $R_{relay}$ in Problem (P1−1) is denoted as $R_{relay}^{*}\left(m_{0}\right)$.

As analyzed above, for a given $m=m_{0}$, Problem (P1−1) must first be solved to obtain the optimal value $R_{relay}^{*}\left(m_{0}\right)$. After that, $R_{relay}^{*}\left(m_{0}\right)$ is compared with $R_{D}^{*}\left(m_{0}\right)$ to derive the solution to Problem (P1): $R_{system}^{*}\left(m_{0}\right)=\max\left\{R_{relay}^{*}\left(m_{0}\right),R_{D}^{*}\left(m_{0}\right)\right\}$. After obtaining the solution to Problem (P1), Problem (P2) is easy to solve since it involves only one variable.

### 3.3. Find the Optimal Solution to Problem (P1−1)

According to Constraint C1−1 in Problem (P1−1), once $\varepsilon_{SR}$ is specified, $\varepsilon_{C}$ becomes $\varepsilon_{C}=\varepsilon_{\max}-\varepsilon_{SR}$. As a result, the problem involves only two variables, $\varepsilon_{SR}$ and η.

**Figure 3:**
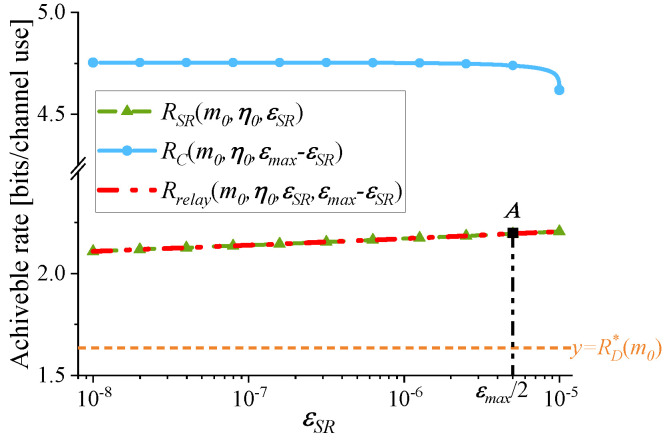
The variation of $R_{SR}$, $R_{C}$, and $R_{relay}$ with respect to $\varepsilon_{SR}$. $m=m_{0}=200$, $\eta =\eta_{0}=0.5$, $\gamma_{SD}=5\;dB$, $\gamma_{SR}=15\;dB$, $\gamma_{RD}=25\;dB$, and $\varepsilon_{max}=10^{-5}$.

**Figure 4:**
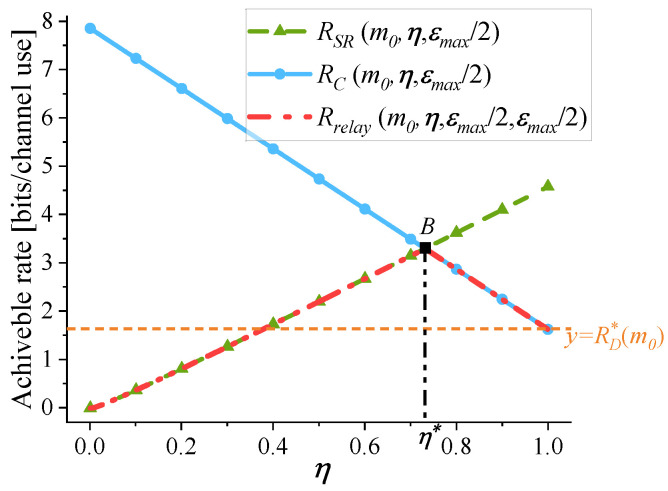
The variation of $R_{SR}$, $R_{C}$, and $R_{relay}$ with respect to η. $m=m_{0}=200$, $\varepsilon_{SR}=\varepsilon_{C}=\varepsilon_{max}/2$, $\gamma_{SD}=5\;dB$, $\gamma_{SR}=15\;dB$, $\gamma_{RD}=25\;dB$, and $\varepsilon_{max}=10^{-5}$.

1. The optimization of $\varepsilon_{SR}$: In existing studies, such as [8,16], near-optimal setting of PEPs, $\varepsilon_{SR}=\varepsilon_{C}=\varepsilon_{max}/2$, is adopted. The effect of this setting is demonstrated in Figure 3 through the observation of a case. The following parameters are used in Figure 3: $\gamma_{SD}=5$ dB, $\gamma_{SR}=15$ dB, $\gamma_{RD}=25$ dB, and $\varepsilon_{max}=10^{-5}$. Given $m=m_{0}=200$, by setting the variable η to $\eta_{0}=0.5$, Figure 3 illustrates the variation of $R_{SR}$, $R_{C}$, and $R_{relay}$ with respect to $\varepsilon_{SR}$. Based on the definition of $R_{relay}$ given in (3), $R_{relay}=\min\left\{R_{SR},R_{C}\right\}=R_{SR}$ holds for any $\varepsilon_{SR}$. In Figure 3, $R_{D}^{*}\left(m_{0}\right)$ is also indicated. As $\varepsilon_{SR}$ approaches $\varepsilon_{max}$, the maximum value of $R_{relay}$ is obtained, which is also the optimal value of $R_{system}$ by comparing $R_{relay}$ with $R_{D}^{*}\left(m_{0}\right)$. At $\varepsilon_{SR}=\frac{\varepsilon_{max}}{2}$ (point *A*), the corresponding $R_{relay}$ deviates from the optimal by just around 0.5%, indicating near-optimal performance. This can be further validated through a mathematical proof, which is shown in Appendix D.
2. The optimization of η: Figure 4 illustrates the variation of $R_{SR}$, $R_{C}$, and $R_{relay}$ with respect to η. Figure 4 adopts the same parameter settings as Figure 3. Given $m=m_{0}=200$, near-optimal setting of PEPs is adopted: $\varepsilon_{SR}=\varepsilon_{C}=\varepsilon_{max}/2$. As shown in Figure 4, when η changes, $R_{relay}$ varies significantly. $R_{relay}$ reaches its maximum at $\eta =\eta^{*}$. When $\eta <\eta^{*}$, the S-R link is the bottleneck link and $R_{relay}=R_{SR}$. When $\eta >\eta^{*}$, $R_{relay}=R_{C}$. Besides, the optimal value of $R_{system}$ in Problem (P1) is also reached at $\eta =\eta^{*}$, because the achievable rate under the RC mode is higher than that under the DC mode.

Based on the above analysis, $R_{relay}$ exhibits large variations with respect to η, but is not sensitive to changes in $\varepsilon_{SR}$. Therefore, to solve Problem (P1−1), the main attention is given to the optimization of η by adopting near-optimal setting of PEPs: $\varepsilon_{SR}=\varepsilon_{C}=\varepsilon_{max}/2$.

The presence of the positive integer Constraint C5−1 increases the computational complexity of solving Problem (P1−1). Therefore, to begin with, Problem (P1−1) is solved without taking Constraint C5−1 into account. Once the optimal solution is obtained, η is modified through rounding ηm, in order to meet Constraint C5−1.

For a given $m_{0}$, $R_{relay}\left(m_{0},\eta ,\varepsilon_{max}/2,\varepsilon_{max}/2\right)$ is limited by the bottleneck link. To achieve the maximum value of $R_{relay}\left(m_{0},\eta ,\varepsilon_{max}/2,\varepsilon_{max}/2\right)$, Proposition 3 indicates η should be optimized to ensure that the achievable rate of the first hop and that of the second hop reach equilibrium.

**Proposition** **3.** *When Constraint C5−1 is temporarily ignored, the maximum value of $R_{relay}\left(m_{0},\eta ,\varepsilon_{max}/2,\varepsilon_{max}/2\right)$ for Problem (P1−1), denoted as $R_{relay}^{w/o\;C5}\left(m_{0}\right)$, is determined by the larger achievable rate of the following two cases. 1) Case 1: substitute $\eta =\eta_{relay,1}^{w/o\;C5}\left(m_{0}\right)=1$ into (3). This is an upper bound and cannot be achieved since η=1 represents the DC mode and $R_{relay}$ loses its physical significance. 2) Case 2: find $\eta_{relay,2}^{w/o\;C5}\left(m_{0}\right)$ that satisfies the following condition for a given $m_{0}$.*

(7) $$\begin{array}{c} R_{SR}\left(m_{0},\eta_{relay,2}^{w/o\;C5}\left(m_{0}\right),\varepsilon_{max}/2\right)=R_{C}\left(m_{0},\eta_{relay,2}^{w/o\;C5}\left(m_{0}\right),\varepsilon_{max}/2\right). \end{array}$$

**Proof of Proposition** **3.** Please see Appendix E.    □

The solution for $\eta_{relay,2}^{w/o\;C5}\left(m_{0}\right)$ in (7) is provided in Proposition 4.

**Proposition** **4.** *Using Ferrari’s method, the $\eta_{relay,2}^{w/o\;C5}\left(m_{0}\right)$ that satisfies (7) is calculated as*

(8) $$\begin{array}{c} \eta_{relay,2}^{w/o\;C5}\left(m_{0}\right)={\left(-\frac{B}{4A}+\frac{W-\sqrt{-(3a+2y+\frac{2b}{W})}}{2}\right)}^{2}. \end{array}$$

*The auxiliary variables a,b,y,A,B,W can be calculated using (A15)–(A23).*

**Proof of Proposition** **4.** Please see Appendix F.    □

The achievable rate under the RC mode in Case 1 and Case 2 are $R_{relay}(m_{0},\eta_{relay,1}^{w/o\;C5}\left(m_{0}\right),$$\varepsilon_{max}/2,\varepsilon_{max}/2)$ and $R_{relay}\left(m_{0},\eta_{relay,2}^{w/o\;C5}\left(m_{0}\right),\varepsilon_{max}/2,\varepsilon_{max}/2\right)$, respectively. According to Proposition 3, $R_{relay}^{w/o\;C5}\left(m_{0}\right)$ is calculated as

(9) $$\begin{array}{c} R_{relay}^{w/o\;C5}\left(m_{0}\right)=\max\{R_{relay}\left(m_{0},\eta_{relay,1}^{w/o\;C5}\left(m_{0}\right),\varepsilon_{max}/2,\varepsilon_{max}/2\right),\\ R_{relay}\left(m_{0},\eta_{relay,2}^{w/o\;C5}\left(m_{0}\right),\varepsilon_{max}/2,\varepsilon_{max}/2\right)\}. \end{array}$$

The corresponding solution is $\left(\eta_{relay}^{w/o\;C5}\left(m_{0}\right),\varepsilon_{max}/2,\varepsilon_{max}/2\right)$, where $\eta_{relay}^{w/o\;C5}\left(m_{0}\right)=1$ if $R_{relay}\left(m_{0},1,\varepsilon_{max}/2,\varepsilon_{max}/2\right)>R_{relay}\left(m_{0},\eta_{relay,2}^{w/o\;C5}\left(m_{0}\right),\varepsilon_{max}/2,\varepsilon_{max}/2\right)$. Otherwise, $\eta_{relay}^{w/o\;C5}\left(m_{0}\right)=\eta_{relay,2}^{w/o\;C5}\left(m_{0}\right)$.

We then take Constraint C5−1 into account. η is modified through rounding ηm. Rounding ηm up will result in $R_{SR}$ being slightly higher than $R_{C}$. $R_{SR}$ will be slightly lower than $R_{C}$ if ηm is rounded down. The choice between rounding ηm up or down is determined by which approach results in a higher $R_{relay}$. Thus, the optimal value of $R_{relay}$ in Problem (P1−1) is

(10) $$\begin{array}{cc} \; & R_{relay}^{*}\left(m_{0}\right)=\\ \; & \max\left\{R_{relay}\left(m_{0},\frac{\left\lceil \eta_{relay}^{w/o\;C5}\left(m_{0}\right)m_{0}\right\rceil }{m_{0}},\varepsilon_{max}/2,\varepsilon_{max}/2\right),\right.\\ \; & \left.R_{relay}\left(m_{0},\frac{\left\lfloor \eta_{relay}^{w/o\;C5}\left(m_{0}\right)m_{0}\right\rfloor }{m_{0}},\varepsilon_{max}/2,\varepsilon_{max}/2\right)\right\}, \end{array}$$

where $\left\lfloor \eta_{relay}^{w/o\;C5}\left(m_{0}\right)m_{0}\right\rfloor$ and $\left\lceil \eta_{relay}^{w/o\;C5}\left(m_{0}\right)m_{0}\right\rceil$ represent the floor function and ceiling function applied to $\eta_{relay}^{w/o\;C5}\left(m_{0}\right)m_{0}$, respectively. The optimal η to Problem (P1−1) is $\eta_{relay}^{*}\left(m_{0}\right)=\frac{\left\lceil \eta_{relay}^{w/o\;C5}\left(m_{0}\right)m_{0}\right\rceil }{m_{0}}$ if $R_{relay}\left(m_{0},\frac{\left\lceil \eta_{relay}^{w/o\;C5}\left(m_{0}\right)m_{0}\right\rceil }{m_{0}},\varepsilon_{max}/2,\varepsilon_{max}/2\right)\geq R_{relay}(m_{0},$$\frac{\left\lfloor \eta_{relay}^{w/o\;C5}\left(m_{0}\right)m_{0}\right\rfloor }{m_{0}},\varepsilon_{max}/2,\varepsilon_{max}/2)$. Otherwise, $\eta_{relay}^{*}\left(m_{0}\right)=\frac{\left\lfloor \eta_{relay}^{w/o\;C5}\left(m_{0}\right)m_{0}\right\rfloor }{m_{0}}$. Then, $(\eta_{relay}^{*}\left(m_{0}\right),$$\varepsilon_{max}/2,\varepsilon_{max}/2)$ is the optimal solution to Problem (P1−1).

### 3.4. Find the Optimal Solution to Problem (P1)

After obtaining the maximum achievable rate of each mode, the maximum value of $R_{system}$ in Problem (P1) is calculated as

(11) $$\begin{array}{c} R_{system}^{*}\left(m_{0}\right)=\max\left\{R_{relay}^{*}\left(m_{0}\right),R_{D}^{*}\left(m_{0}\right)\right\}. \end{array}$$

The corresponding solution to Problem (P1) is $\left(\eta_{system}^{*}\left(m_{0}\right),\varepsilon_{max}/2,\varepsilon_{max}/2\right)$, where $\eta_{system}^{*}\left(m_{0}\right)=\eta_{relay}^{*}\left(m_{0}\right)$ if $R_{relay}^{*}\left(m_{0}\right)>R_{D}^{*}\left(m_{0}\right)$. Otherwise, $\eta_{system}^{*}\left(m_{0}\right)=1$. The proposed algorithm is outlined in Algorithm 1.
**Algorithm 1 **JPB-ARM using Ferrari’s Method (Algorithm for Problem (P1))**Input:** Channel conditions: $\gamma_{SD}$, $\gamma_{SR}$, $\gamma_{RD}$, maximum latency: $\varepsilon_{max}$, total blocklength: $m_{0}\in N^{+}$1:Adopt near-optimal setting of PEPs: $\varepsilon_{SR}=\varepsilon_{C}=\varepsilon_{max}/2$;2:$\eta_{relay,1}^{w/o\;C5}\left(m_{0}\right)=1$, and calculate $\eta_{relay,2}^{w/o\;C5}\left(m_{0}\right)$ by (8);3:Calculate $R_{relay}^{w/o\;C5}\left(m_{0}\right)$ and $\eta_{relay}^{w/o\;C5}\left(m_{0}\right)$ by (9);4:Calculate $R_{relay}^{*}\left(m_{0}\right)$ and $\eta_{relay}^{*}\left(m_{0}\right)$ by (10);5:Calculate $R_{D}^{*}\left(m_{0}\right)$ by substituting $m=m_{0}$ and $\varepsilon_{D}=\varepsilon_{max}$ into (5);6:Calculate $R_{system}^{*}\left(m_{0}\right)$ and $\eta_{system}^{*}\left(m_{0}\right)$ by (11);**Output:** Optimal solution to Problem (P1): $\eta_{system}^{*}\left(m_{0}\right)$, $E_{\mathrm{system}}^{*}\left(m_{0}\right)=$$\left\{\varepsilon_{max},\varepsilon_{max}/2,\varepsilon_{max}/2\right\}$, the optimal objective value of Problem (P1): $R_{system}^{*}\left(m_{0}\right)$

### 3.5. Find the Optimal Solution to Problem (P2)

Problem (P2) exhibits the following properties:

1. Single variable: Problem (P2) involves only one variable, *m*.
2. Monotonicity of $mR_{system}^{*}\left(m\right)$: Based on Proposition 1, it is easy to prove that the information bits that can be transmitted, $mR_{system}^{*}\left(m\right)$, are monotonically increasing with respect to *m*.
3. Existence of a solution: Proposition 1 establishes the condition ensuring solution existence. Due to the monotonicity of $mR_{system}^{*}\left(m\right)$, the existence of a solution implies its uniqueness.
4. Applicability of the bisection algorithm: Let $m_{l}=0$. $R_{system}^{*}\left(m_{l}\right)=0$ because it is impossible to transmit any data when the blocklength is zero. Thus, $m_{l}R_{system}^{*}\left(m_{l}\right)\leq k$. If the solution $m^{*}$ exists, let $m_{u}=m_{max}$. Also, $m_{u}$ satisfies $m_{u}R_{system}^{*}\left(m_{u}\right)\geq k$. Due to the monotonicity of $mR_{system}^{*}\left(m\right)$, $m^{*}$ is located in the range $\left[m_{l},m_{u}\right]$. By iteratively narrowing the interval $\left[m_{l},m_{u}\right]$, convergence to $m^{*}$ is achieved. This aligns with the way the bisection algorithm works.

**Figure 5:**
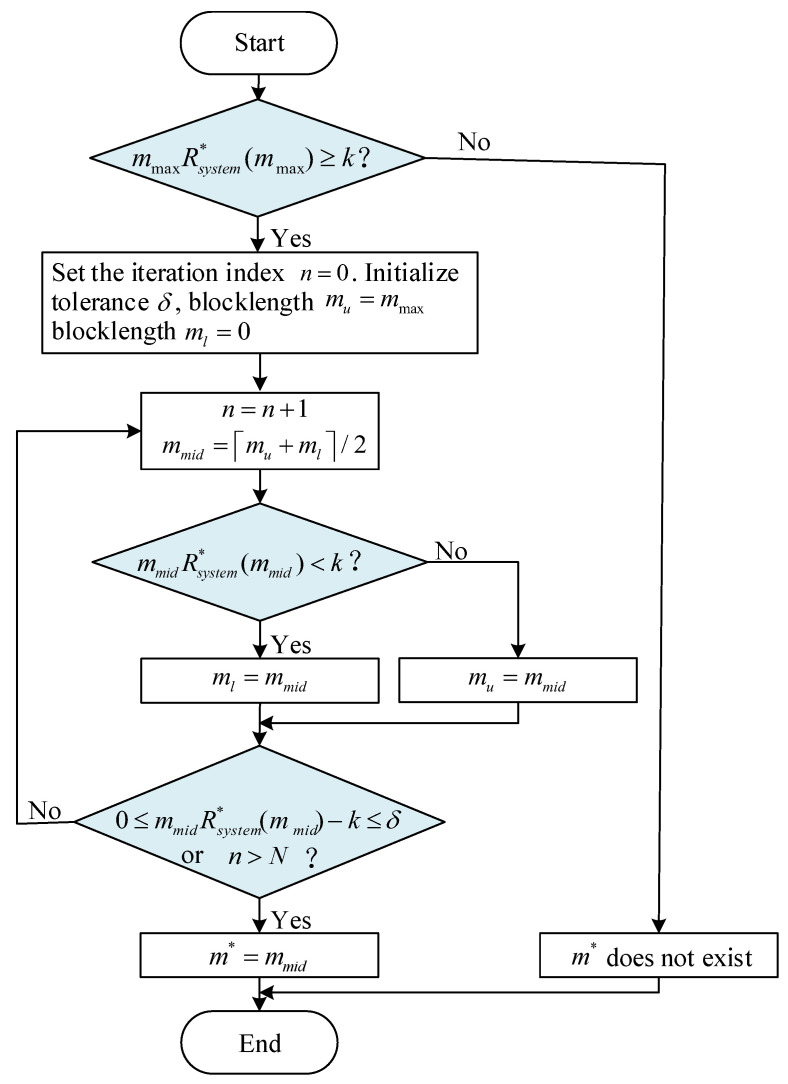
The flowchart of Algorithm 2. The blue-highlighted areas in the flowchart correspond to procedures that rely on Algorithm 1 for calculation.

According to the above analysis, the bisection algorithm is introduced to solve Problem (P2), as outlined in Algorithm 2. To adapt the bisection algorithm to the integer constraint on *m*, we ensure that the interval boundaries remain integers at each iteration. For better readability, a flowchart of Algorithm 2 is presented in Figure 5. The blue-highlighted areas in the flowchart correspond to procedures that rely on Algorithm 1 for calculation, specifically for determining the values of $R_{system}^{*}\left(m_{max}\right)$ and $R_{system}^{*}\left(m_{mid}\right)$.
**Algorithm 2 **RCM Using Bisection Search (Algorithm for (P2))**Input:** Channel conditions: $\gamma_{SD}$, $\gamma_{SR}$, $\gamma_{RD}$, maximum latency: $\varepsilon_{max}$, packet size in bits: *k*, maximum blocklength: $m_{max}$, the maximum iteration count: *N*1:Calculate $R_{system}^{*}\left(m_{max}\right)$ using Algorithm 1;2:**if**$\;m_{max}R_{system}^{*}\left(m_{max}\right)>k\;$**then**3:     Set the iteration index n=0. Initialize tolerance δ, blocklength $m_{u}=m_{max}$, blocklength $m_{l}=0$;4:     **repeat**5:        n=n+1;6:        $m_{mid}=\left\lceil (m_{l}+m_{u})/2\right\rceil$;7:        Calculate $R_{system}^{*}\left(m_{mid}\right)$ using Algorithm 1;8:        **if** $m_{mid}R_{system}^{*}\left(m_{mid}\right)<k$ **then**9:            $m_{l}=m_{mid}$;10:        **else**11:            $m_{u}=m_{mid}$;12:     **until** $0<m_{mid}R_{system}^{*}\left(m_{mid}\right)-k<\delta$ **or** n>N13:     $m^{*}=m_{mid}$;14:**else**15:     **if** $m_{max}R_{system}^{*}\left(m_{max}\right)=k$ **then**16:        $m^{*}=m_{max}$;17:     **else**18:        $m^{*}$ does not exist;**Output:** Optimal solution to Problem (P2): $m^{*}$

## 4. Simulation Results and Analysis

This section presents simulation results to evaluate Algorithms 1 and 2. The scenario depicted in Figure 1 is considered. The power density of additive white Gaussian noise (AWGN) is −174 dBm/Hz. The path loss model in [17] is adopted: (38.46+20×lgd) dB, where *d* denotes the distance between the transmitter and receiver. The comprehensive simulation parameters are detailed in Table 2.

The proposed algorithms are compared with the common relay algorithms in recent studies. Building on the previous analysis presented in the introduction, three common algorithms are considered.

1. Algorithm A-EA-MRC [5]. It assumes the direct link is available (A), and the blocklength is evenly allocated (EA) for each hop. The receiver applies MRC method to merge the data.
2. Algorithm A-EA-MIC [6]. It differs from Algorithm A-EA-MRC by employing the MI combining (MIC) method to merge the data at the receiver.
3. Algorithm NA-OA [10]. It assumes the direct link is not available (NA), and the blocklength is optimally allocated (OA).

In all algorithms, the near-optimal setting of PEPs is adopted.

### 4.1. Performance Evaluation of Algorithm 1

An analysis of the optimal solution and the corresponding objective value for Problem (P1) is presented in this subsection.

For a given value of $m_{0}$, the maximum achievable rate for Algorithm A-EA-MRC, A-EA-MIC, and NA-OA is denoted as $R_{system}^{A-EA-MRC}\left(m_{0}\right)=\max\left(R_{relay}^{A-EA-MRC}\left(m_{0}\right),R_{D}^{*}\left(m_{0}\right)\right)$, $R_{system}^{A-EA-MIC}\left(m_{0}\right)=\max\left(R_{relay}^{A-EA-MIC}\left(m_{0}\right),R_{D}^{*}\left(m_{0}\right)\right)$, and $R_{system}^{NA-OA}\left(m_{0}\right)=R_{relay}^{NA-OA}\left(m_{0}\right)$, respectively. $R_{relay}^{A-EA-MRC}\left(m_{0}\right)$, $R_{relay}^{A-EA-MIC}\left(m_{0}\right)$, and $R_{relay}^{NA-OA}\left(m_{0}\right)$ are the achievable rate under the RC mode for different algorithms. Algorithm NA-OA assumes the direct link is not available. Therefore, the system can only communicate under the RC mode.

**Figure 6:**
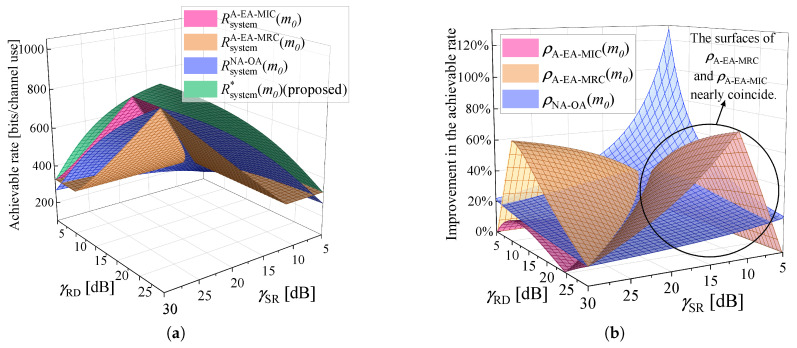
Compare the system’s maximum achievable rate for different algorithms as $\gamma_{SR}$ and $\gamma_{RD}$ vary. $\gamma_{SD}=5$ dB, $\varepsilon_{max}=10^{-5}$, and $m_{0}=200$. (**a**) The maximum achievable rate of the system. (**b**) The improvement of $R_{system}^{*}\left(m_{0}\right)$ over $R_{system}^{A-EA-MRC}\left(m_{0}\right)$, $R_{system}^{A-EA-MIC}\left(m_{0}\right)$, and $R_{system}^{NA-OA}\left(m_{0}\right)$.

Figure 6a illustrates the maximum achievable rate of different algorithms as $\gamma_{SR}$ and $\gamma_{RD}$ vary. Parameter settings in Figure 6: $\gamma_{SD}=5$ dB, $\varepsilon_{max}=10^{-5}$, and $m_{0}=200$. It can be observed that as $\gamma_{SR}$ and $\gamma_{RD}$ vary, the proposed Algorithm 1 always achieves the highest achievable rate. We use $\rho_{Y}\left(m_{0}\right)=\left(\frac{R_{system}^{*}\left(m_{0}\right)-R_{system}^{Y}\left(m_{0}\right)}{R_{system}^{Y}\left(m_{0}\right)}\right)*100\%$, Y∈{A−EA−MRC,A−EA−MIC,NA−OA} to denote the improvement in the achievable rate, $R_{system}^{*}$, for Algorithm 1 over other algorithms. To provide a more intuitive comparison of achievable rate, $\rho_{A-EA-MRC}\left(m_{0}\right)$, $\rho_{A-EA-MIC}\left(m_{0}\right)$, and $\rho_{NA-OA}\left(m_{0}\right)$ are shown in Figure 6b. In Figure 6b, when the difference between $\gamma_{SR}$ and $\gamma_{RD}$ is significant, $\rho_{A-EA-MRC}\left(m_{0}\right)$ and $\rho_{A-EA-MIC}\left(m_{0}\right)$ are high. This means that equal blocklength allocation is appropriate only when channel conditions of the two hops are close. At low $\gamma_{SR}$ or $\gamma_{RD}$, $\rho_{A-EA-MRC}\left(m_{0}\right)$ and $\rho_{A-EA-MIC}\left(m_{0}\right)$ are zero. This is because the DC mode has higher achievable rate than the RC mode, and is chosen by all algorithms. $\rho_{NA-OA}\left(m_{0}\right)$ is always greater than 0, since the direct link is neglected by Algorithm NA-OA. Algorithm 1 has better utilization of links and blocklength resources, and improves average performance of the achievable rate by 32.06%, 20.28%, and 24.80% over A-EA-MRC, A-EA-MIC, and NA-OA, respectively.

**Figure 7:**
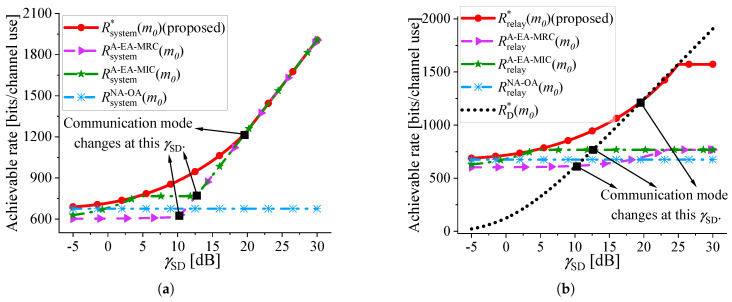
Compare the achievable rate of different algorithms as $\gamma_{SD}$ varies. $\gamma_{SR}=25$ dB, $\gamma_{RD}=20$ dB, $\varepsilon_{max}=10^{-5}$, and $m_{0}=200$. (**a**) The maximum achievable rate of the system. (**b**) The achievable rate under different communication modes.

To further explain why Algorithm 1 exhibits superior performance, given $\gamma_{SR}=25$ dB and $\gamma_{RD}=20$ dB, $\gamma_{SD}$ is varied to observe the achievable rate of the system in Figure 7a. Furthermore,, the achievable rate under different mode is shown in Figure 7b to help explain the reason behind the change in the achievable rate of the system. Parameter settings in Figure 7: $\varepsilon_{max}=10^{-5}$, and $m_{0}=200$. In Figure 7a, black squares are used to mark the points of communication mode change in Algorithm A-EA-MRC, A-EA-MIC, and Algorithm 1. At the points of change, from left to right, the corresponding $\gamma_{SD}$ is denoted as $\gamma_{SD}^{A-EA-MRC}$, $\gamma_{SD}^{A-EA-MIC}$, and $\gamma_{SD}^{*}$, respectively. These points are also shown in Figure 7b. The communication mode of Algorithm NA-OA does not change, because the system can only communicate under the RC mode. In Figure 7b, it can be seen that $R_{D}^{*}\left(m_{0}\right)$ intersects with $R_{system}^{A-EA-MRC}\left(m_{0}\right)$, $R_{system}^{A-EA-MIC}\left(m_{0}\right)$, and $R_{system}^{*}\left(m_{0}\right)$ at $\gamma_{SD}^{A-EA-MRC}$, $\gamma_{SD}^{A-EA-MIC}$, and $\gamma_{SD}^{*}$, respectively. For each algorithm, the RC mode is chosen in the region preceding the turning point on its respective curve. In Figure 7b, when $\gamma_{SD}<\gamma_{SD}^{*}$, Algorithm 1 uses the RC mode, and $R_{relay}^{*}\left(m_{0}\right)$ is always the highest compared with Algorithm A-EA-MRC and A-EA-MIC. Because the blocklength allocation is optimized by Algorithm 1 to ensure that the achievable rate of the first hop and that of the second hop are equal, optimal resource utilization is achieved. In comparison, the performance of Algorithm A-EA-MRC and A-EA-MIC is limited by the bottleneck link. When $\gamma_{SD}>\gamma_{SD}^{*}$, Algorithm 1, A-EA-MRC, and A-EA-MIC all use the DC mode, resulting in equal achievable rates. Although blocklength optimization is addressed in Algorithm NA-OA, it assumes that the direct link is not available, which is only valid at low $\gamma_{SD}$. Therefore, when $\gamma_{SD}$ is high, the achievable rate of Algorithm NA-OA is the lowest.

**Figure 8:**
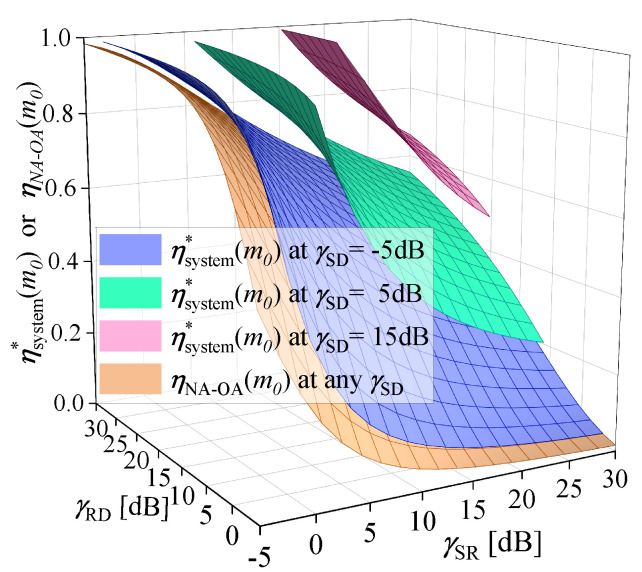
$\eta_{system}^{*}\left(m_{0}\right)$ and $\eta_{NA-OA}\left(m_{0}\right)$ under changing $\gamma_{SR}$ and $\gamma_{RD}$ at given $\gamma_{SD}$. $\varepsilon_{max}=10^{-5}$, and $m_{0}=200$.

Since blocklength optimization is considered in Algorithm 1 and Algorithm NA-OA, the optimal blocklength allocation coefficient (OBAC) of them is compared in Figure 8. Parameter settings in Figure 8: $\varepsilon_{max}=10^{-5}$, and $m_{0}=200$. In Algorithm NA-OA, the OBAC is denoted as $\eta_{NA-OA}\left(m_{0}\right)\in \left(0,1\right)$, which means $m_{0}\eta_{NA-OA}\left(m_{0}\right)$ channel uses are allocated to S. At given $\gamma_{SD}$, the surfaces of $\eta_{system}^{*}\left(m_{0}\right)$ and $\eta_{NA-OA}\left(m_{0}\right)$ under different $\gamma_{SR}$ and $\gamma_{RD}$ are illustrated. To facilitate observation, Figure 8 only shows the points with $\eta_{system}^{*}\left(m_{0}\right)<1$ (RC mode). Outside the surface, where $\gamma_{SR}$ or $\gamma_{RD}$ decreases, $\eta_{system}^{*}\left(m_{0}\right)=1$.

In Figure 8, $\eta_{system}^{*}\left(m_{0}\right)$ varies under different channel conditions. When the SNR of other links remains constant, $\eta_{system}^{*}\left(m_{0}\right)$ decreases with increasing $\gamma_{SR}$ and increases with either $\gamma_{SD}$ or $\gamma_{RD}$. This is because the better the channel conditions in each transmission phase, the fewer resources are required. $\eta_{NA-OA}\left(m_{0}\right)$ shows similar patterns of variation. However, $\eta_{NA-OA}\left(m_{0}\right)$ cannot perceive changes in $\gamma_{SD}$, and has similar values to $\eta_{system}^{*}\left(m_{0}\right)$ at low $\gamma_{SD}$, where the direct link can be reasonably ignored. By observing any surface of $\eta_{system}^{*}\left(m_{0}\right)$, it can be found that only when $\gamma_{SR}>\gamma_{SD}$ and $\gamma_{RD}>\gamma_{SD}$ may the RC mode be chosen.

**Figure 9:**
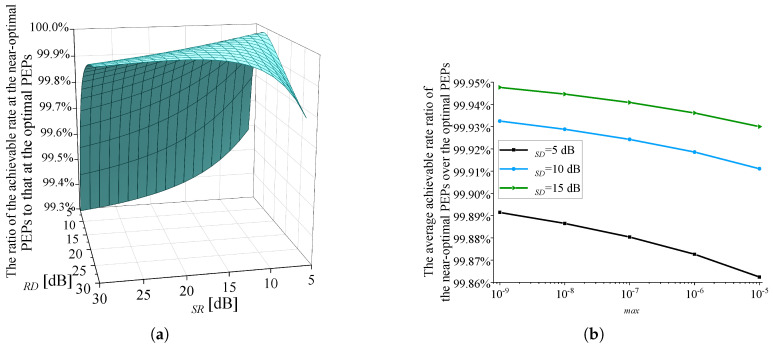
The ratio of the achievable rate achieved at the near-optimal PEPs (Algorithm 1) to that achieved at the optimal PEPs (the exhaustive search approach). $\gamma_{SD}=5$ dB, $m_{0}=200$. (**a**) The change in the ratio of the achievable rate with varying $\gamma_{SR}$ and $\gamma_{RD}$. $\varepsilon_{max}=10^{-5}$. (**b**) The variation of the average achievable rate ratio with respect to $\varepsilon_{max}$.

When solving Problem (P1), Algorithm 1 adopts near-optimal setting of PEPs: $\varepsilon_{SR}=\varepsilon_{C}=\varepsilon_{max}/2$. To evaluate the precision of the near-optimal setting, in Figure 9, a comparison is conducted on the objective function of Problem (P1) under the optimal and near-optimal settings of PEPs. The optimal solution of PEPs can be obtained by using an exhaustive search approach. The exhaustive search approach enumerates all possible allocations by partitioning $\varepsilon_{max}$ into 1% intervals: $\varepsilon_{SR}=\frac{k}{100}\varepsilon_{max},\varepsilon_{C}=\varepsilon_{max}-\varepsilon_{SR},k\in \left\{1,2,...,99\right\}$. Then the optimal $\left(\varepsilon_{SR},\varepsilon_{C}\right)$ that maximizes the objective function of Problem (P1) is selected. Therefore, the exhaustive search approach requires approximately 100 times more computation time than the proposed Algorithm 1. Parameter settings in Figure 9: $\gamma_{SD}=5$ dB, and $m_{0}=200$. In Figure 9a, $\varepsilon_{max}$ is set to $10^{-5}$, and the ratio of the achievable rate achieved at the near-optimal PEPs to that achieved at the optimal PEPs is shown. It can be seen that when $\gamma_{SR}$ and $\gamma_{RD}$ take certain values, the achievable rate ratio reaches 100%, which means a uniform PEP allocation between the two hops is optimal in this case. For other values of $\gamma_{SR}$ and $\gamma_{RD}$, the achievable rate loss caused by the near-optimal PEPs does not exceed 1%. On the surface illustrated in Figure 9a, 40,000 points are evenly selected, and their z-values are averaged. Then, the average achievable rate ratio is 99.86%, which confirms the accuracy of the near-optimal solution of PEPs. Figure 9b illustrates the variation of the average achievable rate ratio with respect to $\varepsilon_{max}$ under different values of $\gamma_{SD}$. It is observed that the achievable rate loss caused by the near-optimal PEPs decreases with decreasing $\varepsilon_{max}$ and increasing $\gamma_{SD}$.

### 4.2. Performance Evaluation of Algorithm 2

An analysis of the optimal solution to Problem (P2) is presented in this subsection.

**Figure 10:**
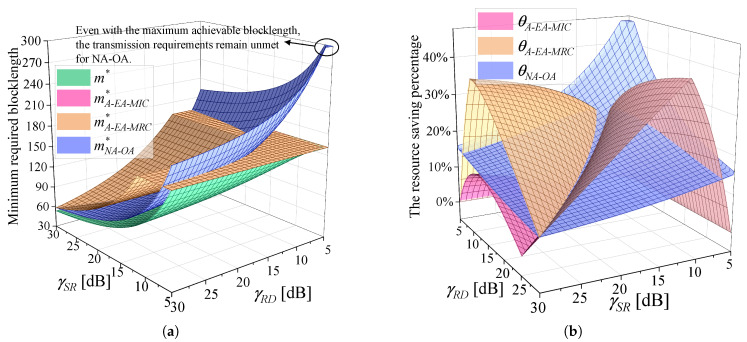
The comparison of the minimum blocklength required by various algorithms. $\gamma_{SD}=5$ dB, $\varepsilon_{max}=10^{-5}$, and k=32 bytes. (**a**) Minimum required blocklength. (**b**) The resource saving percentage of the proposed algorithm over the others.

With the parameter settings $\gamma_{SD}=5$ dB, $\varepsilon_{max}=10^{-5}$, and k=32 bytes, Figure 10a illustrates the minimum blocklength required by various algorithms to satisfy the uRLLC requirements. The total blocklength required by the benchmark algorithms for transmitting *k* bytes is denoted by $m^{Y}$, Y∈{A−EA−MRC,A−EA−MIC,NA−OA}. $m^{*}$ is the minimum blocklength required by the proposed algorithm. As observed, the surface representing the proposed algorithm remains the lowest among all, demonstrating its superiority in minimizing resource consumption under varying $\gamma_{SR}$ and $\gamma_{RD}$. When $\gamma_{SR}$ and $\gamma_{RD}$ are small, the blocklength required by NA-OA reaches $m_{max}$. In fact, even with the maximum achievable blocklength, the transmission requirements remain unmet for NA-OA, which means the constraints cannot be satisfied simultaneously. A joint observation of Figure 6a and Figure 10a reveals that the required blocklength decreases as the achievable rate increases. We use $\theta_{Y}=\left(1-\frac{m^{*}}{m^{Y}}\right)*100\%$, Y∈{A−EA−MRC,A−EA−MIC,NA−OA} to denote the resource saving percentage of the proposed algorithm over the others. To provide a more intuitive comparison of resource saving, $\theta_{A-EA-MRC}$, $\theta_{A-EA-MIC}$, and $\theta_{NA-OA}$ are shown in Figure 10b. Figure 6b and Figure 10b exhibit highly similar trends, and the surface variations observed in Figure 10b can be attributed to the same underlying factors as those in Figure 6b. Compared with Algorithm A-EA-MRC, A-EA-MIC, and NA-OA, the proposed algorithm achieves average resource savings of 21.40%, 14.03%, and 17.18%, respectively.

### 4.3. Application Insights and Discussions

This work introduces a novel PEP and blocklength allocation scheme designed for relay-assisted IIoT applications. Firstly, a near-optimal solution for the PEPs of each hop is derived, and its accuracy is thoroughly validated through both theoretical analysis and simulation results (Figure 9). Secondly, under the RC mode, by flexibly allocating blocklength based on the current channel conditions (Figure 8), the proposed scheme ensures that the achievable rate of the first hop and that of the second hop reach equilibrium. This effectively eliminates the performance limitations imposed by the bottleneck link on the system, achieving more efficient resource utilization compared to Algorithm A-EA-MRC and A-EA-MIC. Besides, in contrast to Algorithm NA-OA, our model does not neglect the impact of the direct communication link. All communication links are effectively utilized. These innovations significantly improve the system available rate under the RC mode (Figure 6 and Figure 7). Accordingly, the proposed scheme demonstrates outstanding performance in minimizing resource consumption under given uRLLC constraints (Figure 10).

Relay-assisted communication is particularly well-suited for IIoT scenarios, where large machinery and equipment may obstruct direct links, as it significantly improves channel quality. Besides, the increasing demand for high transmission quality and the dense deployment of devices have led to a shortage of wireless resources, necessitating improved resource utilization and reduced resource consumption. Therefore, the proposed scheme holds significant practical value.

This work adopts certain simplified assumptions to better highlight the core ideas and demonstrate the performance potential of the proposed scheme. Below, we discuss potential applications and extensions of the proposed scheme in real IIoT scenarios.

- Multi-user scenarios: The scheme introduced in this paper is applicable to multi-user systems, as it allows the decomposition of multi-user transmissions into multiple transmitter–relay–receiver pairs shown in Figure 1, permitting distributed optimization of resource allocation per pair.
- Variable packet sizes, dynamic traffic: The packet size serves as an input parameter to the proposed algorithms. Therefore, the proposed algorithms are capable of operating under various packet sizes and producing the corresponding optimized two-hop blocklength allocation and PEP results. This adaptability enables the algorithms to handle varying traffic conditions in practical IIoT scenarios.
- Interference scenarios: When interference exists, the calculation for channel dispersion changes. The dispersion derived for non-Gaussian noise can be found in [18]. However, the analysis process remains unchanged.
- Dynamic industrial environments (e.g., with mobility): This scenario requires accurate prediction or real-time feedback of channel and network conditions to ensure timely and effective adaptation of resource allocation strategies. Since the proposed scheme can handle PEP and blocklength allocation under arbitrary channel conditions, its performance largely depends on the accuracy of real-time channel condition prediction or feedback. This research direction will be a focus of our future work.
- Multi-relay scenarios: In multi-relay systems, one of the key challenges is how to select the optimal relay. Building on the current research, it is possible to evaluate and predict the communication performance of various relay choices. This can offer valuable guidance for relay selection, which is a meaningful direction for future work.

## 5. Conclusions

In IIoT systems, blockages and fading degrade communication reliability, while the increasing demand for uRLLC further exacerbates wireless resource shortages. To address these challenges, relay-assisted communication is adopted, and the problem of resource consumption minimization is investigated by jointly optimizing the blocklength and PEP of each hop. The original problem is decomposed into two equivalent subproblems. For the first subproblem, Algorithm 1 adopts a uniform PEP allocation between the two hops. Its near-optimality is confirmed through both theoretical analysis and simulation validation. The blocklength is then optimized to equalize the achievable rates of the two hops, and a closed-form solution is derived. For the second subproblem, Algorithm 2 employs a bisection method to minimize the total blocklength. Simulation results confirm that the proposed algorithms significantly reduce resource consumption while satisfying uRLLC requirements. The performance advantages over baseline algorithms highlight the practical potential of the proposed algorithms in resource-constrained industrial environments.

## Figures and Tables

**Table 1:** Summary of main variables.

Variable	Meaning
$\gamma_{SD},\gamma_{SR},\gamma_{RD}$	SNR of each link
$\varepsilon_{SR},\varepsilon_{C},\varepsilon_{D}$	PEP of the corresponding link
$E=\{\varepsilon_{D},\varepsilon_{SR},\varepsilon_{C}\}$	The set of PEPs
ηm	Blocklength occupied by the source node
(1−η)m	Blocklength occupied by the relay node
$R_{SR}\left(m,\eta ,\varepsilon_{SR}\right)$ or $R_{SR}$	The achievable rate of the S-R link
$R_{C}\left(m,\eta ,\varepsilon_{C}\right)$ or $R_{C}$	The achievable rate of the combined link
$R_{relay}\left(m,\eta ,\varepsilon_{SR},\varepsilon_{C}\right)$ or $R_{relay}$	The achievable rate under the RC mode
$R_{D}\left(m,\varepsilon_{D}\right)$ or $R_{D}$	The achievable rate under the DC mode
$R_{system}\left(m,\eta ,E\right)$ or $R_{system}$	The system’s achievable rate under the best communication mode
$R_{relay}^{*}\left(m_{0}\right)$	Maximum achievable rate under the RC mode at $m=m_{0}$
$R_{D}^{*}\left(m_{0}\right)$	Maximum achievable rate under the DC mode at $m=m_{0}$
$R_{system}^{*}\left(m_{0}\right)$	$\max\{R_{system}^{*}\left(m_{0}\right),R_{D}^{*}\left(m_{0}\right)\}$
$m^{*}$	The minimum required total blocklength

**Table 2:** Simulation parameters.

Parameter	Value
The maximum blocklength $m_{max}$	300 channel uses
PEP limit $\varepsilon_{max}$	$10^{-9}∼10^{-5}$
Packet size	32 bytes
White noise power density	−174 dBm/Hz
Path loss model	(38.46+20×lgd)dB
Tolerance δ	0.01
The maximum iteration count *N*	100

**Figure A1:**
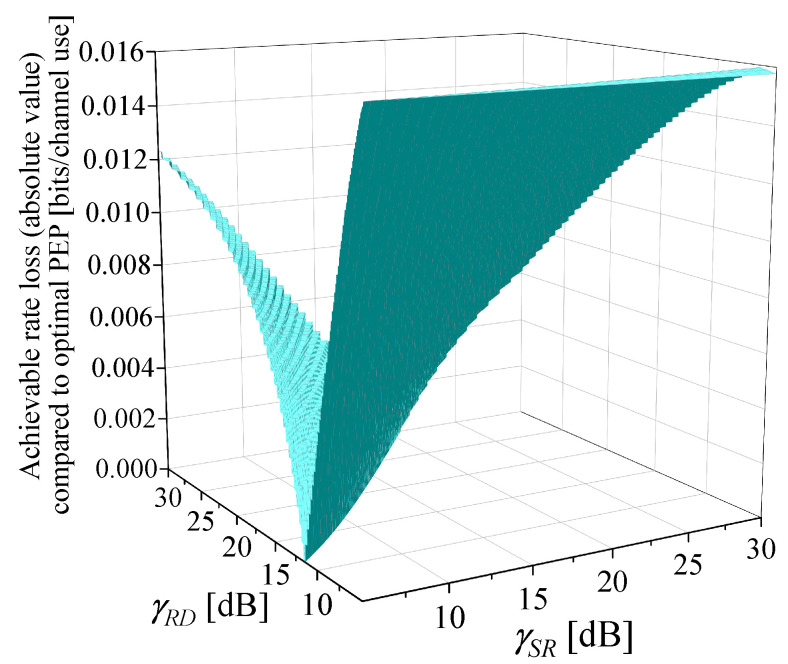
The variation of $\left|\Delta R_{relay}\right|$ with respect to $\gamma_{SR}$ and $\gamma_{RD}$.

## Data Availability

The data presented in this study are available on request from the corresponding author.

## Appendix A. Proof of Proposition 1

Part 1: Firstly, we prove that $R_{system}^{*}\left(m\right)$ is monotonically increasing with respect to *m*.

The first derivative of $R_{SR}\left(m,\eta ,\varepsilon_{SR}\right)$ with respect to *m* is given by

(A1) $$\begin{array}{c} \frac{\partial R_{SR}}{\partial m}=\frac{1}{2}\frac{\sqrt{\eta V_{SR}}Q^{-1}\left(\varepsilon_{SR}\right)}{In2}m^{-\frac{3}{2}}>0. \end{array}$$

Therefore, $R_{SR}\left(m,\eta ,\varepsilon_{SR}\right)$ increases monotonically with respect to *m*. By applying a similar approach, the same conclusion holds for $R_{C}$ and $R_{D}$. Thus, according to (3) and (6), it is easy to prove that $R_{system}\left(m,\eta ,E\right)$ is monotonically increasing in *m*.

Let us consider two possible values for *m*, namely $m_{1}$ and $m_{2}$. Assume that $m_{2}>m_{1}$. We need to prove that $R_{system}^{*}\left(m_{2}\right)>R_{system}^{*}\left(m_{1}\right)$. In Problem (P1), when $m=m_{1}$, the optimal solution is denoted as $\eta_{system}^{*}\left(m_{1}\right)$ and $E_{\mathrm{system}}^{*}\left(m_{1}\right)$. When $m=m_{2}$, the optimal solution is denoted as $\eta_{system}^{*}\left(m_{2}\right)$ and $E_{\mathrm{system}}^{*}\left(m_{2}\right)$. Then, the following inequality holds.

(A2) $$\begin{array}{cc} \; & \;\;\;R_{system}^{*}\left(m_{2}\right)\\ \; & =R_{system}\left(m_{2},\eta_{system}^{*}\left(m_{2}\right),E_{\mathrm{system}}^{*}\left(m_{2}\right)\right)\\ \; & \overset{\left(a\right)}{\geq }R_{system}\left(m_{2},\eta_{system}^{*}\left(m_{1}\right),E_{\mathrm{system}}^{*}\left(m_{1}\right)\right)\\ \; & \overset{\left(b\right)}{>}R_{system}\left(m_{1},\eta_{system}^{*}\left(m_{1}\right),E_{\mathrm{system}}^{*}\left(m_{1}\right)\right)\\ \; & =R_{system}^{*}\left(m_{1}\right). \end{array}$$

The inequality (a) holds, since given $m=m_{2}$, the system’s achievable rate is maximized at $\eta =\eta_{system}^{*}\left(m_{2}\right)$ and $E=E_{\mathrm{system}}^{*}\left(m_{2}\right)$. For other values of η and E, such as $\eta =\eta_{system}^{*}\left(m_{1}\right)$ and $E=E_{\mathrm{system}}^{*}\left(m_{1}\right)$, the achievable rate is no greater than $R_{system}\left(m_{2},\eta^{*}\left(m_{2}\right),E^{*}\left(m_{2}\right)\right)$. The inequality (b) is conducted based on the monotonic increase of $R_{system}\left(m,\eta ,E\right)$ with respect to *m*, as discussed above. This completes the proof of the monotonicity of $R_{system}^{*}\left(m\right)$.

Part 2: Based on Part 1, it is easy to prove that the information bits that can be transmitted, $mR_{system}^{*}\left(m\right)$, is monotonically increasing with respect to *m*. If $m_{max}R_{system}^{*}\left(m_{max}\right)\geq k$, the Problem (P1) and (P2) are feasible. Now, we prove that the optimal solution to Problem (P1) and (P2) is also the optimal solution to Problem (P0).

Assume $m^{\prime }$ is a feasible solution to Problem (P2). In Problem (P1), the optimal solutions corresponding to $m^{\prime }$ are $\eta_{system}^{*}\left(m^{\prime }\right)$ and $E_{\mathrm{system}}^{*}\left(m^{\prime }\right)$. Let X denote the set of all feasible solution pairs $\left(m^{\prime },\eta_{system}^{*}\left(m^{\prime }\right),E_{\mathrm{system}}^{*}\left(m^{\prime }\right)\right)$ that jointly satisfy the constraints of Problems (P1) and (P2), and let Y denote the set of all feasible solution pairs $\left(m^{\prime \prime },\eta^{\prime \prime },E^{\prime \prime }\right)$ to Problem (P0). It is straightforward to verify that (1) any solution in X satisfies Problem (P0)’s constraints, hence X⊆Y; and (2) any solution in Y satisfies the constraints of both Problem (P1) and (P2), hence Y⊆X. Then, X=Y. Therefore, the optimal solution to Problem (P1) and (P2) is also the optimal solution to Problem (P0).

## Appendix B. Proof of Proposition 2

Proposition 2 can be proved by using contradiction.

For a given value of *m*, $m=m_{0}$, suppose that the optimal solution of Problem (P1) is $\left(\eta_{system}^{*}\left(m_{0}\right),E_{\mathrm{system}}^{*}\left(m_{0}\right)\right)$, and Constraint C1 is satisfied with a strict inequality at $\left(\eta_{system}^{*}\left(m_{0}\right),E_{\mathrm{system}}^{*}\left(m_{0}\right)\right)$. $E_{system}^{*}\left(m_{0}\right)=\left\{\varepsilon_{SR}^{*},\varepsilon_{RD}^{*},\varepsilon_{D}^{*}\right\}$. According to Constraint C1, if $\eta_{system}^{*}\left(m_{0}\right)=1$, $\varepsilon_{D}^{*}$ should be less than $\varepsilon_{max}$. Then, a new solution $\varepsilon_{D}^{\prime }$ can be found that satisfies $\varepsilon_{D}^{*}<\varepsilon_{D}^{\prime }\leq \varepsilon_{max}$. $R_{D}$ defined in (5) are monotonically increase with $\varepsilon_{D}$, which is easy to prove. Therefore, $R_{D}\left(m_{0},\varepsilon_{D}^{\prime }\right)$ is higher than $R_{D}\left(m_{0},\varepsilon_{D}^{*}\right)$. This is a contradiction. If $0<\eta_{system}^{*}\left(m_{0}\right)<1$, by applying the same proof technique, an identical conclusion can be drawn.

## Appendix C

For a given value of $m_{0}$, to obtain the solution to Problem (P1), Problem (P1−1) should be first solved to determine the maximum achievable rate under the RC mode: $R_{relay}^{*}\left(m_{0}\right)$. Then, $R_{relay}^{*}\left(m_{0}\right)$ is compared with the achievable rate under the DC mode. Therefore, extending the feasible domain of η may change the optimal solution to (P1−1), thereby affecting the solution to Problem (P1). However, extending the feasible domain of η from (0,1) to (0,1] in Problem (P1−1) does not alter the solution of Problem (P1). A theoretical analysis is conducted to explain this.

If $R_{relay}^{*}\left(m_{0}\right)$ is obtained at η<1, clearly, the solution to Problem (P1−1) and (P1) will not be affected. Next, we analyze the situation where $R_{relay}^{*}\left(m_{0}\right)$ is obtained at η=1. In Problem (P1), since $\varepsilon_{SR}>0,\varepsilon_{C}>0$, and $\varepsilon_{SR}+\varepsilon_{C}\;\;=\varepsilon_{\max}$ are required in the RC mode, and $\varepsilon_{D}=\varepsilon_{max}$ is required in the DC mode, we have $\varepsilon_{C}<\varepsilon_{\max}=\varepsilon_{D}$ and $Q^{-1}\left(\varepsilon_{C}\right)>Q^{-1}\left(\varepsilon_{D}\right)$. If $R_{relay}^{*}\left(m_{0}\right)$ is obtained at η=1, $R_{relay}\left(m_{0},1,\varepsilon_{SR},\varepsilon_{C}\right)=\min${$R_{SR}\left(m_{0},1,\varepsilon_{SR}\right),R_{C}\left(m_{0},1,\varepsilon_{C}\right)$}$\;\leq R_{C}\left(m_{0},1,\varepsilon_{C}\right)$. Substituting η=1 into (2) and comparing it with (5) yields $R_{C}\left(m_{0},1,\varepsilon_{C}\right)<R_{D}^{*}\left(m_{0}\right)$. Therefore, the optimal value $R_{system}^{*}\left(m_{0}\right)$ of Problem (P1) is equal to $R_{D}^{*}\left(m_{0}\right)$, not $R_{relay}^{*}\left(m_{0}\right)$. Then, the DC mode is chosen. Obviously, this result is the same when η∈(0,1).

## Appendix D

According to (3), to establish that $R_{relay}$ varies little between the near-optimal and exact optimal $\varepsilon_{SR}$, it is necessary to demonstrate that $R_{SR}$ and $R_{C}$ are similarly close at these two points. Given $m=m_{0}$, denote the optimal solution to Problem (P1−1) as $\varepsilon_{SR}^{*}$ and $\eta^{*}$. Using [19]

(A3) $$\begin{array}{c} \frac{d}{d\varepsilon_{SR}}Q^{-1}\left(\varepsilon_{SR}\right)=-\sqrt{2\pi }e^{\frac{{\left[Q^{-1}\left(\varepsilon_{SR}\right)\right]}^{2}}{2}}, \end{array}$$

at $\left(m=m_{0},\eta =\eta^{*},\varepsilon_{SR}=\varepsilon_{SR}^{*}\right)$, the partial derivative of $R_{SR}\left(m,\eta ,\varepsilon_{SR}\right)$ with respect to $\varepsilon_{SR}$ can be obtained as

(A4) $$\begin{array}{c} \frac{\partial R_{SR}}{\partial \varepsilon_{SR}}\left|(m_{0},\eta^{*},\varepsilon_{SR}^{*})\right.=\sqrt{\frac{2\pi \eta^{*}V_{SR}}{m_{0}}}\frac{1}{\ln2}e^{\frac{{\left[Q^{-1}\left(\varepsilon_{SR}^{*}\right)\right]}^{2}}{2}}. \end{array}$$

When $\varepsilon_{SR}$ changes from $\varepsilon_{SR}^{*}$ to $\varepsilon_{max}/2$, the variation in $\varepsilon_{SR}$ is $\Delta \varepsilon_{SR}=\varepsilon_{max}/2-\varepsilon_{SR}^{*}$. It is evident that $\left|\Delta \varepsilon_{SR}\right|\leq \varepsilon_{max}$, where $\left|\cdot \right|$ denotes the process of computing the absolute value. Reference [2] indicates that $\varepsilon_{max}$ is typically below $10^{-5}$, which is sufficiently small. Therefore, the corresponding variation in $R_{SR}$ can be approximated by $\Delta \varepsilon_{SR}\frac{\partial R_{SR}}{\partial \varepsilon_{SR}}\left|(m_{0},\eta^{*},\varepsilon_{SR}^{*})\right.$. By employing a similar method, at $\left(m_{0},\eta^{*},\varepsilon_{SR}^{*}\right)$, the partial derivative of $R_{C}\left(m,\eta ,\varepsilon_{C}\right)=R_{C}\left(m,\eta ,\varepsilon_{max}-\varepsilon_{SR}\right)$ with respect to $\varepsilon_{SR}$ can be obtained as

(A5) $$\begin{array}{c} \frac{\partial R_{C}}{\partial \varepsilon_{SR}}\left|(m_{0},\eta^{*},\varepsilon_{SR}^{*})\right.=-\sqrt{\frac{2\pi V_{C}}{m_{0}}}\frac{1}{\ln2}e^{\frac{{\left[Q^{-1}\left(\varepsilon_{max}-\varepsilon_{SR}^{*}\right)\right]}^{2}}{2}}. \end{array}$$

When $\varepsilon_{SR}$ changes from $\varepsilon_{SR}^{*}$ to $\varepsilon_{max}/2$, the corresponding variation in $R_{C}$ can be approximated by $\Delta \varepsilon_{SR}\frac{\partial R_{C}}{\partial \varepsilon_{SR}}\left|(m_{0},\eta^{*},\varepsilon_{SR}^{*})\right.$.

According to Proposition 3, at $\left(m_{0},\eta^{*},\varepsilon_{SR}^{*}\right)$, $R_{SR}=R_{C}$. Thus, if $\Delta \varepsilon_{SR}>0$, $\Delta \varepsilon_{SR}\frac{\partial R_{SR}}{\partial \varepsilon_{SR}}\left|(m_{0},\eta^{*},\varepsilon_{SR}^{*})\right.>0$, while $\Delta \varepsilon_{SR}\frac{\partial R_{C}}{\partial \varepsilon_{SR}}\left|(m_{0},\eta^{*},\varepsilon_{SR}^{*})\right.<0$. Thus, at $\left(m_{0},\eta^{*},\varepsilon_{max}/2\right)$, $R_{SR}>R_{C}$. In this case, the combined link is the bottleneck link, which determines the achievable rate of the system. Accordingly, the loss of the system rate, $\Delta R_{relay}$, is $\Delta \varepsilon_{SR}\frac{\partial R_{C}}{\partial \varepsilon_{SR}}\left|(m_{0},\eta^{*},\varepsilon_{SR}^{*})\right.$. Likewise, if $\Delta \varepsilon_{SR}<0$, $\Delta R_{relay}=\Delta \varepsilon_{SR}\frac{\partial R_{SR}}{\partial \varepsilon_{SR}}\left|(m_{0},\eta^{*},\varepsilon_{SR}^{*})\right.$.

Different channel conditions lead to different values of $\Delta R_{relay}$. Given $\gamma_{SD}=5$ dB, Figure A1 illustrates the variation of $\left|\Delta R_{relay}\right|$ with respect to $\gamma_{SR}$ and $\gamma_{RD}$. Figure A1 presents the values of $\left|\Delta R_{relay}\right|$ for $\gamma_{SR}>5$ dB and $\gamma_{RD}>5$ dB. Because, if $\gamma_{SR}<\gamma_{SD}$ or $\gamma_{RD}<\gamma_{SD}$, the RC mode will not be selected, and the optimization of $\varepsilon_{SR}$ is no longer meaningful. As shown in Figure A1, when $\gamma_{SR}$ and $\gamma_{RD}$ take certain values, $\left|\Delta R_{relay}\right|=0$, which means $\varepsilon_{SR}=\varepsilon_{max}/2$ is optimal in this case. As $\gamma_{SR}$ or $\gamma_{RD}$ decreases, $\left|\Delta R_{relay}\right|$ increases gradually. Nevertheless, $\left|\Delta R_{relay}\right|$ is consistently much smaller than 1. Therefore, the precision of the near-optimal PEP is verified.

## Appendix E. Proof of Proposition 3

In Proposition 3, Problem (P1−1) is considered without Constraint C5−1. Given $m=m_{0}$, $\varepsilon_{SR}=\varepsilon_{max}/2$, and $\varepsilon_{C}=\varepsilon_{max}/2$, $R_{SR}$, $R_{C}$, and $R_{relay}$ are functions of a single variable η. We extend the domain of η in $R_{SR}\left(m_{0},\eta ,\varepsilon_{max}/2\right)$, $R_{C}\left(m_{0},\eta ,\varepsilon_{max}/2\right)$, and $R_{relay}\left(m_{0},\eta ,\varepsilon_{max}/2,\varepsilon_{max}/2\right)$ to [0,1]. $R_{relay}\left(m_{0},\eta ,\varepsilon_{max}/2,\varepsilon_{max}/2\right)$ is continuous on the interval [0,1] with respect to η, since $R_{SR}\left(m_{0},\eta ,\varepsilon_{max}/2\right)$ and $R_{C}\left(m_{0},\eta ,\varepsilon_{max}/2\right)$ are continuous. Based on the extreme value theorem, $R_{relay}\left(m_{0},\eta ,\varepsilon_{max}/2,\varepsilon_{max}/2\right)$ has a maximum value on the interval [0,1].

To find the global maximum value of $R_{relay}\left(m_{0},\eta ,\varepsilon_{max}/2,\varepsilon_{max}/2\right)$, we can compare all local maximum values at critical points, which may be the local maximum values of $R_{SR}\left(m_{0},\eta ,\varepsilon_{max}/2\right)$ or $R_{C}\left(m_{0},\eta ,\varepsilon_{max}/2\right)$, the boundary values and the intersection points of $R_{SR}\left(m_{0},\eta ,\varepsilon_{max}/2\right)$ and $R_{C}\left(m_{0},\eta ,\varepsilon_{max}/2\right)$. Next, the values that are not possible are excluded. The second derivatives of $R_{SR}\left(m_{0},\eta ,\varepsilon_{max}/2\right)$ and $R_{C}\left(m_{0},\eta ,\varepsilon_{max}/2\right)$ are

(A6) $$\begin{array}{c} \frac{d^{2}R_{SR}\left(m_{0},\eta ,\varepsilon_{max}/2\right)}{d\eta^{2}}=\frac{1}{4}\eta^{-\frac{3}{2}}\sqrt{\frac{V_{SR}}{m}}\frac{Q^{-1}\left(\varepsilon_{max}/2\right)}{\ln2}, \end{array}$$

and

(A7) $$\begin{array}{c} \frac{d^{2}R_{C}\left(m_{0},\eta ,\varepsilon_{max}/2\right)}{d\eta^{2}}=\frac{1}{4}{\left(\frac{\eta V_{SD}+\left(1-\eta \right)V_{RD}}{m}\right)}^{-\frac{3}{2}}\\ {\left(\frac{V_{SD}-V_{RD}}{m}\right)}^{2}\frac{Q^{-1}\left(\varepsilon_{max}/2\right)}{\ln2}. \end{array}$$

It can be seen that $\lim_{\eta \to 0^{+}}\frac{d^{2}R_{SR}\left(m_{0},\eta ,\varepsilon_{max}/2\right)}{d\eta^{2}}=+\infty$. $\frac{d^{2}R_{SR}\left(m_{0},\eta ,\varepsilon_{max}/2\right)}{d\eta^{2}}>0$ and $\frac{d^{2}R_{C}\left(m_{0},\eta ,\varepsilon_{max}/2\right)}{d\eta^{2}}>0$ on [0,1]. Since the second derivative should be less than or equal to zero at a local maximum, $R_{SR}\left(m_{0},\eta ,\varepsilon_{max}/2\right)$ and $R_{C}\left(m_{0},\eta ,\varepsilon_{max}/2\right)$ do not have local maximum values. Next, observing the boundary values, it can be found that

(A8) $$\begin{array}{cc} \; & R_{relay}\left(m_{0},1,\varepsilon_{max}/2,\varepsilon_{max}/2\right)\\ & =\min\{R_{SR}\left(m_{0},1,\varepsilon_{max}/2\right),R_{C}\left(m_{0},1,\varepsilon_{max}/2\right)\}\\ & >\min\{R_{SR}\left(m_{0},0,\varepsilon_{max}/2\right),R_{C}\left(m_{0},0,\varepsilon_{max}/2\right)\}=0. \end{array}$$

Therefore, the global maximum value of $R_{relay}\left(m_{0},\eta ,\varepsilon_{max}/2,\varepsilon_{max}/2\right)$ on the interval [0,1] can only occur at η=1 or intersection points of $R_{SR}\left(m_{0},\eta ,\varepsilon_{max}/2\right)$ and $R_{C}\left(m_{0},\eta ,\varepsilon_{max}/2\right)$, which is also the global maximum value on the interval (0,1].

## Appendix F. Proof of Proposition 4

Let $x=\sqrt{\eta }$. By substituting (1)–(2) into (7), (7) can be rewritten as

(A9) $$\begin{array}{c} a_{0}x^{2}+b_{0}x-c_{0}{\left(V_{SD}x^{2}+V_{RD}\left(1-x^{2}\right)\right)}^{\frac{1}{2}}+d_{0}=0, \end{array}$$

where

(A10) $$\begin{array}{cc} \; & a_{0}=C_{SD}-C_{SR}-C_{RD} \end{array}$$

(A11) $$\begin{array}{cc} \; & b_{0}=\sqrt{\frac{V_{SR}}{m_{0}}}\frac{Q^{-1}\left(\varepsilon_{max}/2\right)}{\ln2} \end{array}$$

(A12) $$\begin{array}{cc} \; & c_{0}=\sqrt{\frac{1}{m_{0}}}\frac{Q^{-1}\left(\varepsilon_{max}/2\right)}{\ln2} \end{array}$$

(A13) $$\begin{array}{cc} \; & d_{0}=C_{RD}. \end{array}$$

Move the $c_{0}{\left(V_{SD}x^{2}+V_{RD}\left(1-x^{2}\right)\right)}^{\frac{1}{2}}$ term to the right-hand side of (A9), and then square both sides. A quartic equation in *x* is obtained by performing this operation, which can be expressed as

(A14) $$\begin{array}{c} Ax^{4}+Bx^{3}+Cx^{2}+Dx+E=0, \end{array}$$

where $A={a_{0}}^{2},B=2a_{0}b_{0},C=2a_{0}d_{0}+{b_{0}}^{2}+{c_{0}}^{2}\left(V_{RD}-V_{SD}\right),D=2b_{0}d_{0},E={d_{0}}^{2}-{c_{0}}^{2}V_{RD}.$

Ferrari’s method [20] is employed to obtain the analytic solutions, as it can achieve high-precision solutions to quartic equations. First, calculate the auxiliary variables a,b,c,P,Q,R,U,y,W using the following equations:

(A15) $$\begin{array}{cc} \; & a=-\frac{3B^{2}}{8A^{2}}+\frac{C}{A} \end{array}$$

(A16) $$\begin{array}{cc} \; & b=\frac{B^{3}}{8A^{3}}-\frac{BC}{2A^{2}}+\frac{D}{A} \end{array}$$

(A17) $$\begin{array}{cc} \; & c=-\frac{3B^{4}}{256A^{4}}+\frac{CB^{2}}{16A^{3}}-\frac{BD}{4A^{2}}+\frac{E}{A} \end{array}$$

(A18) $$\begin{array}{cc} \; & P=-\frac{a^{2}}{12}-c \end{array}$$

(A19) $$\begin{array}{cc} \; & Q=-\frac{a^{3}}{108}+\frac{ac}{3}-\frac{b^{2}}{8} \end{array}$$

(A20) $$\begin{array}{cc} \; & R=-\frac{Q}{2}-\sqrt{\frac{Q^{2}}{4}+\frac{P^{3}}{27}} \end{array}$$

(A21) $$\begin{array}{cc} \; & U=\sqrt[{3}]{R} \end{array}$$

(A22) $$\begin{array}{cc} \; & y=-\frac{5a}{6}+\left\{\begin{array}{c} U=0\;\to -\sqrt[{3}]{Q}\\ U\neq 0\;\to U-\frac{P}{3U} \end{array}\right. \end{array}$$

(A23) $$\begin{array}{cc} \; & W=\sqrt{a+2y}. \end{array}$$

It is important to note that the square root, $c_{0}{\left(V_{SD}x^{2}+V_{RD}\left(1-x^{2}\right)\right)}^{\frac{1}{2}}$, has been squared when transitioning from (A9) to (A14). Squaring is a non-reversible operation, which may result in an expanded solution set. Therefore, the solutions of (A14) must be substituted back into (A9) to eliminate any extraneous solutions that do not satisfy the original equation. After verification, the solutions to (A9) can be obtained. Since $x=\sqrt{\eta }$, the solutions to (7) is given in (8).
